# The plant transcriptome—from integrating observations to models

**DOI:** 10.3389/fpls.2013.00048

**Published:** 2013-03-11

**Authors:** Björn Usadel, Alisdair R. Fernie

**Affiliations:** ^1^Institute for Biology 1, RWTH Aachen UniversityAachen, Germany; ^2^IBG-2, Pflanzenwissenschaften, Forschungszentrum JülichJülich, Germany; ^3^Max-Planck-Institute of Molecular Plant PhysiologyPotsdam, Germany

Transcriptomes as assessed by either microarrays or next-generation sequencing have produced a hitherto unprecedented data flood regarding transcript identity and levels in plant systems. Microarray data has been extensively used over the last 15 years or so and evaluation of the data thus produced has progressed well beyond early statistically quality evaluation and descriptive lists to a mature science whereby gene networks and cascades have been able to provide mechanistic insight. The development of sensitive quantitative PCR for lowly expressed genes such as transcription factors has additionally allowed another layer of complexity to be accessed and the modeling of transcription factor expression with that of target genes has met considerable success. Yet more recently, data emanating from RNAseq studies have greatly improved the coverage of transcript profiling. That said, this technology further compounded transcriptome analysis by making it possible to identify differentially spliced transcripts etc. In this research topic we would like to provide an “on the fly” portrait of the use of either microarray or RNAseq based datasets in contemporary Plant Systems Biology.

Given the relative simplicity of doing so, much information has been gleaned from microarray datasets by assuming guilt-by-association. The success of this approach is summarized by articles of Provart (2012) and Tohge and Fernie (2012), as are recent studies that go beyond transcription and link in physiological and metabolic aspects. As in the legal process from which the approach lifts its name it is important to note that suspects obtained this way require “fair trial” since assuming “guilt” is fraught with dangers as summarized in Usadel et al. (2009a). Thus, Tohge and Fernie extend the use of the co-expression approach for the annotation of assumed gene function and discuss bringing in further experimental “evidence” as provided by metabolomics, proteomics, or physiological measurements (Tohge et al., 2005; De Boldt et al., 2012). They then delve further into the subject by explaining how to make a more solid case by linking gene functions across multiple species (Mutwil et al., 2011; Obayashi et al., 2011). The review by Provart (2012) also reviews novel aspects of visualized correlations, however, pays more attention to marrying these data with subcellular localization and tissue/organ specific networks such as those defined by SeedNet (Kohl et al., 2011) and the overlay of such networks with those derived from protein-protein interaction studies (Geisler-Lee et al., 2007).

Junker et al. (2012a) follow a similar direction extending on ideas put forward in their recent Trends in Biotechnology review (Junker et al., 2012b) here focusing their attention on visual analysis of the transcriptome. They provide an overview of plant transcriptomics repositories and detail how these can serve as useful resources for visualization programs such as HIVE as well as detailing how the color-coded output from such programs can be integrated with known biological networks using analysis of floral homeotic gene expression patterns and seed expression profiles as exemplary case studies. They further discuss information visualization standards as suggested by Card et al. (1999) and the eFP browser (Winter et al., 2007). Friedel et al. (2012) and Grene et al. (2012) follow a similar approach whereby they re-analyse data using both visualization and network techniques both interested in abiotic conditions. Whereas Friedel uses network approaches and functional categories to investigate stress responses, Grene focuses on winter hardening in spruce. Interestingly Grene et al. (2012) is able to show a reprogramming of the cell wall and nucleotide sugar metabolism using MapMan (Usadel et al., 2009b) and GO ontologies.

However, when it comes to data analysis of whole genome expression datasets, particularly those obtained from complex temporally and/or spatially resolved experiments visualization helps in finding “the meaning within the noise.” Thus, currently the researcher typically zooms in on a particular subset of the data which excites their biological curiosity, often obtaining such data from public repositories such as genevestigator (https://www.genevestigator.com/gv). But much information and potentially knowledge is untapped by adopting this approach. This leaves one wondering if aided by modern biostatistics and bioinformatics one shouldn't be able to do better. To improve this situation Klie et al. (2012) present a computational solution wherein recent extension of the principal component analysis variants STATIS and dual-STATIS (Lavit et al., 1994; Abdi et al., 2012) is applied to study the time resolved response of Arabidopsis thaliana to perturbations in the prevailing light and/or temperature conditions. This proof-of-concept study illustrates that these tools can clearly aid in dataset-wide analyses and furthermore that they can specify the extent to which either the transcript levels or alternatively the experimental treatments reflect these perturbations thus providing biological insight across the entire datasets obtained.

As is evident from the multitude of manuscripts dealing with microarray data, there is still much to be learned from these data sets. However, time moves on and whilst it seems difficult to teach old dogs new omics tricks, RNAseq is slowly becoming more and more popular. Already machine learning techniques are trickling in to help separating noise from the data. Thus, Thieme et al. (2012) try to find the proverbial needle in the haystack by identifying Argonaute sorting signals for miRNAs. Whilst mutual information didn't indicate any other than the 5′ position to dictate which of the 10 Argonaute proteins is processing which miRNA, Thieme solve the problem of having only four possible 5′ bases for 10 different proteins, by showing that other positions likely play a role as well.

Such analyses are assuming, however, that one actually knows which transcripts to deal with. But one of the perceived beauties of RNAseq is that one could learn about the transcriptome on the fly whilst analysing the data by assembling the reads into transcripts. This seems, however, an ambituous goal and thus in their article Schliesky et al. (2012) address the question RNAseq assembly—are we there yet? They review plant applications of 454/Roche and Illumina sequencing which have in combination, to date, already been used to assess the transcriptome of over 50 plant species. Although they argue these approaches have been useful in downstream applications such as proteomics (Lopez-Casado et al., 2012) and the same can be argued for their recent use to augment recent genome sequencing efforts (Tomato Genome Consortium, 2012), assemblies may well not accurately reflect the actual plant transcriptomes, especially if not checked well. In order to ameliorate challenges for the transciptome assembly problem they provide a list of quality control parameters and the necessary scripts to produce them most likely providing an invaluable resource for this burgeoning area of transcriptomes and bringing the old idea of genomeless genomics (Rudd, 2005) within the reach of even the smallest labs.

Rose et al. (2012) then round up the uses of RNAseq by providing both insights into how RNAseq has already benefited the plant communityand detailed examples where genomeless genomics was used. Extending beyond this, they show that RNAseq is also valuable in finding small non-coding RNA highlighting the manner demonstrated in the Thieme et al. (2012) article. In addition they demonstrate how important RNAseq can be for bulk segregant analysis and thus the identification of causal mutations. Alongside these illustrations they additionally provide the wet bench biologist with comprehensive workflows on how the RNA should be processed for these varied applications.

Finally, in his article Kliebenstein (2012), tries to answer the other burning questions of RNA-seq—How deep does deep-sequencing need to go to capture the majority of network or genomic information present in a variety of transciptomics experiments? To address this question he applied Shannon entropy analysis to existing Arabidopsis transcriptomics data namely a co-expression network, an expression QTL analysis and a temporal analysis of the circadian clock. Intriguingly, he came to the conclusion that at least 80% of the information present in a transcriptomic study is likely obtainable by measuring only the top 10% of the transcripts within a sample. This, rather surprising, finding has important consequences for experimental design particularly with concern to the scale and affordability of large-scale studies.

## References

[B1] Abdi H. Williams L. J. Valentin D. Bennani-Dosse M. (2012). STASIS and DISTATIS: optimum multi-table principal component analysis and three way metric multidimensional scaling. Wiley Interdiscipl. Rev. Comput. Stat. 4, 124–167

[B2] Card S. K. Mackinlay J. D. Shneiderman B. (eds). (1999). Readings in Information Visualization: Using Vision to Think. San Francisco, CA: Morgan Kaufmann Publishers Inc.

[B3] De Boldt S. Hollunder J. Nelisen H. Meulemeester N. Inze D. (2012). CORNET 2.0: integrating plant co-expression, protein-protein interactions, regulatory interactions, gene associations and functional associations. New Phytol. 195, 707–720 10.1111/j.1469-8137.2012.04184.x 22651224

[B4] Friedel S. Usadel B. Von Wiren N. Sreenivasulu N. (2012). Reverse engineering. Key component of systems biology to unravel global abiotic stress cross-talks. Front. Plant Sci. 3:294 10.3389/fpls.2012.00294 23293646 PMC3533172

[B5] Geisler-Lee J. O'Toole N. Ammar R. Provart N. J. Millar A. H. Geisler M. (2007). A predicted interactome for Arabidopsis. Plant Physiol. 145, 317–329 10.1104/pp.107.103465 17675552 PMC2048726

[B6] Grene R. Klumas C. Suren H. Yang K. Collakova E. Myers E. S. (2012). Mining and visualization of microarray and metabolomic data reveal extensive cell wall remodeling during winter handening in *Sitka spruce* (*Picea sitchensis*). Front. Plant Sci. 3:241 10.3389/fpls.2012.00241 23112803 PMC3482696

[B7] Junker A. Rohn H. Schreiber F. (2012a). Visual analysis of transcriptomic data in the context of anatomical structures and biological networks. Front. Plant Sci. 3:252 10.3389/fpls.2012.00252 23162564 PMC3498740

[B8] Junker A. Sorokin A. Czauderna T. Schreiber F. Mazein A. (2012b). Wiring diagrams in biology: towards the standardized representation of biological information. Trends Biotechnol. 30, 555–557 10.1016/j.tibtech.2012.08.003 22979995

[B9] Klie S. Caldana C. Nikoloski Z. (2012). Compromise of multiple time-resolved transciptomics experiments identifies tightly regulated functions. Front. Plant Sci. 3:249 10.3389/fpls.2012.00249 23162561 PMC3499770

[B10] Kliebenstein D. J. (2012). Exploring the shallow end; estimating information content in transcriptomic studies. Front. Plant Sci. 3:213 10.3389/fpls.2012.00213 22973290 PMC3437520

[B11] Kohl M. Wiese S. Warscheid B. (2011). Cytoscape: software for visualisation and analysis of biological networks. Methods Mol. Biol. 696, 291–303 10.1007/978-1-60761-987-1_18 21063955

[B12] Lavit C. Escoufier Y. Sabatier R. Traissac P. (1994). The, ACT (STASIS method). Computational 18, 97–119

[B13] Lopez-Casado G. Covey P. A. Bedinger P. A. Mueller L. A. Thannhauser T. W. Zhang S. (2012). Enabling proteomic studies with RNA-Seq: the proteome of tomato pollen as a test case. Proteomics 12, 761–774 10.1002/pmic.201100164 22539427

[B14] Mutwil M. Klie S. Tohge T. Giorgi F. M. Wilkins O. Campbell M. (2011). PlaNet: combined sequence and expression comparisons across plant networks derived from seven species. Plant Cell 23, 895–910 10.1105/tpc.111.083667 21441431 PMC3082271

[B15] Obayashi T. Nishida K. Kasahara K. Kinoshita K. (2011). ATTED-II updates: condition specific gene coexpression to extend coexpression analyses and applications to a broad range of flowering plants. Plant Cell Physiol. 52, 213–219 10.1093/pcp/pcq203 21217125 PMC3037081

[B16] Provart N. J. (2012). Correlation networks visualization. Front. Plant Sci. 3:240 10.3389/fpls.2012.00240 23181065 PMC3500995

[B17] Rose J. K. C. Martin L. Fei Z. Giovannoni J. J. (2012). Catalyzing plant science research with RNA-seq. Front. Plant Sci. [Accepted]. 10.3389/fpls.2013.00066 PMC3612697 23554602

[B18] Rudd S. (2005). OpenSputnik - a database to ESTablish comparative plant genomics using unsaturated sequence collections. Nucleic Acids Res. 33, 622–627 10.1093/nar/gki040 15608275 PMC539994

[B19] Schliesky S. Gowik U. Weber A. P. M. Bräutigam A. (2012). RNA-seq assembly – Are we there yet? Front. Plant Sci. 3:220 10.3389/fpls.2012.00220 23056003 PMC3457010

[B20] Thieme C. J. Schudoma C. May P. Walther D. (2012). Give it AGO: the search for miRNA-Argonaute sorting signals in Arabidopsis thaliana indicates a relevance of sequence positions other than the 5′-position alone. Front. Plant Sci. 3:272 10.3389/fpls.2012.00272 23233858 PMC3516803

[B21] Tohge T. Fernie A. R. (2012). Co-expression and co-response within and beyond transcription. Front. Plant Sci. 3:248 10.3389/fpls.2012.00248 23162560 PMC3492870

[B22] Tohge T. Nishiyama Y. Hirai M. Y. Yano M. Nakajima J. Awazuhara M. (2005). Functional genomics by integrated genomics analysis of metabolome and transciptome of Arabidopsis plants over-expressing an MYB transcription factor. Plant J. 42, 218–235 10.1111/j.1365-313X.2005.02371.x 15807784

[B23] Tomato Genome Consortium. (2012). The tomato genome sequence provides insights into fleshy fruit evolution. Nature 485, 635–641 10.1038/nature11119 22660326 PMC3378239

[B24] Usadel B. Obayashi T. Mutwil M. Giorgi F. M. Bassel G. W. Tanimoto M. (2009a). Coexpression tools for plant biology: opportunities for hypothesis generation and caveats. Plant Cell Environ. 32, 1633–1651 10.1111/j.1365-3040.2009.02040.x 19712066

[B25] Usadel B. Poree F. Nagel A. Lohse M. Czedik-Eysenberg A. Stitt M. (2009b). A guide to using MapMan to visualize and compare Omics data in plants: a case study in the crop species, Maize. Plant Cell Environ. 32, 1211–1229 10.1111/j.1365-3040.2009.01978.x 19389052

[B26] Winter D. Vinegar B. Nahal H. Ammar R. Wilson G. V. Provart N. J. (2007). An “electronic fluroescent pictogram” browser for exploring and analyzing large-scale biological data sets. PLoS ONE 8:e718 10.1371/journal.pone.0000718 17684564 PMC1934936

